# A chemical perspective on high pressure crystal structures and properties

**DOI:** 10.1093/nsr/nwz144

**Published:** 2019-10-01

**Authors:** John S Tse

**Affiliations:** Department of Physics and Engineering Physics, University of Saskatchewan, Saskatoon, Saskatchewan S7N 5E2, Canada

**Keywords:** high pressure, crystal structure, chemical bonding, superconductivity

## Abstract

The general availability of third generation synchrotron sources has ushered in a new era of high pressure research. The crystal structure of materials under compression can now be determined by X-ray diffraction using powder samples and, more recently, from multi-nano single crystal diffraction. Concurrently, these experimental advancements are accompanied by a rapid increase in computational capacity and capability, enabling the application of sophisticated quantum calculations to explore a variety of material properties. One of the early surprises is the finding that simple metallic elements do not conform to the general expectation of adopting 3D close-pack structures at high pressure. Instead, many novel open structures have been identified with no known analogues at ambient pressure. The occurrence of these structural types appears to be random with no rules governing their formation. The adoption of an open structure at high pressure suggested the presence of directional bonds. Therefore, a localized atomic hybrid orbital description of the chemical bonding may be appropriate. Here, the theoretical foundation and experimental evidence supporting this approach to the elucidation of the high pressure crystal structures of group I and II elements and polyhydrides are reviewed. It is desirable and advantageous to extend and apply established chemical principles to the study of the chemistry and chemical bonding of materials at high pressure.

## INTRODUCTION

Pressure is an important and versatile thermodynamic variable to alter the structure and chemical bonding of a material. It is now routine to vary the pressure in the laboratory by many orders of magnitude (compared to temperature), e.g. from ambient (bar) to Mbar, using the diamond anvil cell [1]. In conjunction with advances in spectroscopic and scattering techniques, mostly based on synchrotron radiation, it is now possible to characterize the properties of picolitre-sized samples under extreme temperature and pressure conditions [2,3]. Originally, high-pressure science was mainly within the domain of physicists, used to study changes in the electronic properties of highly compressed materials. Later, high pressure techniques became staple tools of Earth and planetary scientists investigating the nature of minerals in the Earth’s mantle and giant planets [4]. In chemistry, there was initial interest in employing pressure in the study of chemical kinetics to validate the activation volume theory [5]. However, the field was dormant from late 1960  as there was no feasible experimental means to significantly increase pressure.

A revolutionary change in high pressure science was made in the 70–80s with the introduction of the diamond anvil cell and new designs of high pressure presses together with the emergence and availability of intense synchrotron radiation sources [6]. Compression using opposing diamond anvils on a sample surrounded in a gasket provides a relatively simple means to generate very high pressure. Moreover, focused and high-brilliance tunable-energy synchrotron X-radiation together with new detection techniques have extended the application of conventional spectroscopic and diffraction methods for *in situ* characterization of material properties [6]. This breakthrough was highlighted in a series of structural studies on the high pressure polymorphs of simple metallic elements in late 1990s and early 2000s in which new and unanticipated crystal structures were reported [7]. Very often, the structural types found have no correspondence to existing structures at ambient pressure and seem to defy explanation based on well-established concepts of chemical bonding. Moreover, exotic properties are often found to associate with these novel structures. The property is related to both the electronic and the crystalline structure; thus the chemistry is significantly altered by pressure. Most notable is the prediction and verification of superconductivity with a very high critical temperature (*T*_c_) in dense metallic hydrides. In fact, very high *T*_c_ has been reported in highly compressed superconducting hydrogen sulfide (200 K) [8] and lanthanum hydride (260 K) [9]. Other recent examples are the formation of crystalline structures between rare gas and hydrogen [10–17], the reactions of hydrogen with nitrogen [18] and silica [19], metal alloys formed from immiscible liquids [20,21] and many others, all occurring at a relatively low pressure of a few 10s of gigapascals. These observations should also have a significant impact on geochemistry as chemical reactions occurring in the Earth’s mantle and core are under high pressure and temperature conditions that may not be adequately described from the knowledge of chemical principles under ambient conditions. These surprising experimental findings ushered in a new era in structural chemistry and the investigation of the structure–property relationship of high pressure compounds. The challenging question is whether research at high pressure can be guided by established practical chemical principles, similar to those developed over the years for ambient chemistry.

This review focuses primarily on the effect of pressure on electronic structure and chemical bonding. I will discuss structural changes and properties in the framework of atomic orbital hybridizations and interactions between frontier orbitals. This contribution is not intended to be a comprehensive survey of the field of high pressure chemistry and physics. Readers are referred to several recent reviews covering the state-of-the-art and different aspects of high pressure science [22–24]. This article serves to illustrate how conventional bonding concepts can be adapted to the understanding of the structures and structural transformations at high pressure with examples drawn mainly on the experience of the author. The development of an orbital-based theory for high pressure is still in the early stage. Arguments presented here may appear to be qualitative. Further refinement of these ideas is required. The motive of this contribution is to stimulate readers to further pursue experimental and theoretical investigations in this direction.

## ATOMS UNDER COMPRESSION

The concept of directional chemical bonds and the prediction of crystal structures of elemental solids is based on the hybridization between valence and low-lying empty orbitals of atoms and the number of electrons available for chemical bonding [25,26]. The latter is directly related to the atomic number and, therefore, to the position of the element in the periodic table [27]. In the periodic table, elements are arranged in Groups according to the number of valence electrons. Elements in the same group of the periodic table share similar chemical properties. Elements in the first two Groups (I and II) are the alkali and alkaline metals with one and two valence electrons in the outermost *s* orbitals, respectively. The first ionization energies of Group I and II elements are comparatively low and susceptible to ionic bonding. The low ionization energies are due to the effective shielding of the nucleus charge by core electrons. Towards the right of the periodic table, the valence *s* orbitals are fully occupied and the valence *p* orbitals are filled gradually from pnictogens (Group XV, N, P, As, …), chalcogens (Group XVI, O, S, Se, …), halogens (Group XVII, F, Cl, Br, …) and finally the noble gases (Group XVIII, He, Ne, Ar, Kr, …). A distinctive group of elements occupying the period table is the transition metals. Those are elements with *d* electrons in the outermost shell. Heavier elements with *d* and *f* electrons in the valence orbitals are called lanthanides and actinides.

A very useful general rule to determine the spatial extent of the valence orbital is the number of nodes of the radial hydrogenic-like valence orbital wave function (*n* − *l* − 1), where *n* is the principal quantum number and *l* the azimuthal quantum number. For example, the 1 *s* orbital of an H atom (*n* = 1, *l* = 0) has no radial node; therefore, the potential energy felt by the valence electron from the nuclear charge is not screened and the ionization energy is 13.6 eV. Except for the rare gas elements with completely filled valence shells, it is the highest among all elements. In comparison, the outermost 6 *s* orbital of the Cs atom has five radial nodes and 54 core electrons (*n* = 6, *l* = 0). Thus the 6 *s* electron is well shielded from the nuclear charge and the first ionization potential is only 3.89 eV, the lowest of all elements. Consequently, the spatial extension of the Cs 6 *s* orbital is fairly diffuse. Adding to the volume occupied by the core electrons, the Cs atomic radius of 3.34 Å is very large. For first row elements, there are no radial nodes in the valence 2 *p* orbitals and, therefore, they are not well screened (*n* = 2, *l* = 1). Thus, the radial extent (size of the atom) of a 2*p* orbital becomes smaller as the nuclear charge increases. A similar effect is also present in the first-row transition metals (*n* = 3 and *l* = 2) where the shielding is poor and the 3*d* orbitals are contracted (tight). In general, the atomic radius increases down a group and from right to left across the same row in the periodic table. The periodic trend of the elements assists in determining the atomic size and provides a useful rule-of-thumb guidance to the activity of the outermost electrons toward chemical bonding.

**Figure 1.**
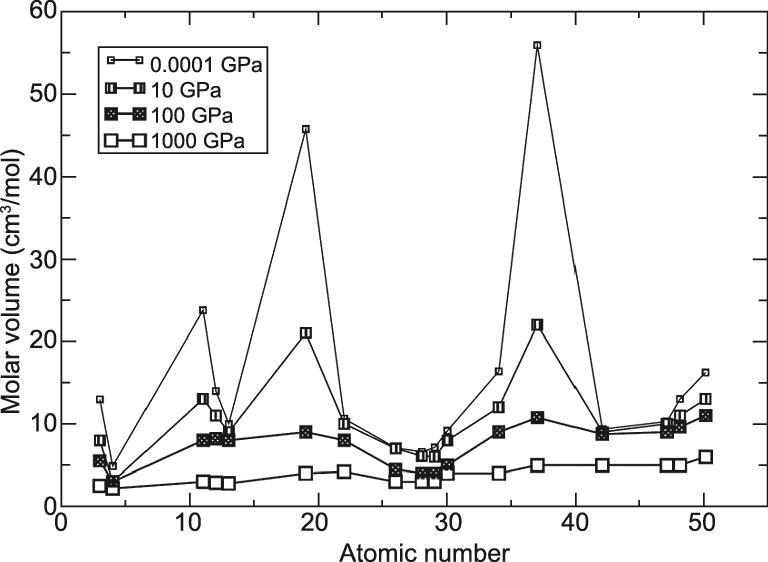
Schematic on the change of atomic size with applied pressure (from Tse and Boldyerva [29]).

What happens to the periodic trends when atoms are compressed? The change in the atomic size under pressure from ambient (1 bar) to 1000 GPa (10 Mbar) is illustrated schematically in Fig. 1 [28,29]. At ambient pressure, atomic sizes of the Group I alkali metals are much larger than other elements of the same row. As pressure increases, the atoms are compacted and their sizes become more uniform. At 100 GPa (1 Mbar), there is no notable difference in the sizes of all (alkali and transition metal) elements [28,29]. The qualitative rules of periodic chemical trend disappear.

**Figure 2.**
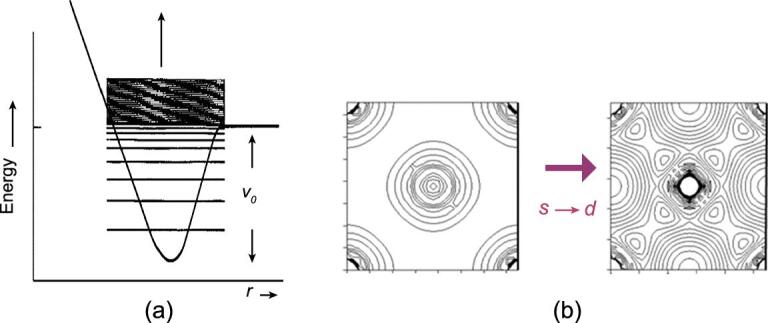
(a) Energy levels of a particle in a potential well and (b) an illustration of the *s–d* orbital hybridization of Cs under pressure.

How are we going to predict chemical bonding at high pressure? The first clue was provided by Sternheimer on the mixing of the valence with low lying empty orbitals [30]. To explain the volume discontinuity in solid Cs observed by Bridgeman at 4.5 GPa, he suggested that the Cs 6*s* band mixed (hybridized) with the empty 5*d* band. This suggestion is supported by band structure calculations [30]. The transfer of an electron from the diffuse 6*s* to ‘tighter or more compact’ 5*d* orbitals resulted in a smaller effective size for the Cs atom and led to a volume reduction at the structural phase transition. Alternatively, the result can be understood from the viewpoint of a simple ‘particle-in-a-box’ (PIB) model. In the PIB, the energy of the electron levels is inversely proportional to the inverse square of the width of the potential well. Upon compression, the width decreases and the energy levels are then pushed higher. In Cs, the empty 5*d* orbitals are ‘trapped’ by the atomic centrifugal potential (−*l*(*l* + 1)/*r*^2^), a phenomenon known as ‘shape resonance’ in atomic spectroscopy (Fig. 2a) [31]. The near degeneracy between 5*d* and 6*s* facilitates *s* → *d* mixing (hybridization). The change from the predominantly ‘*s*’ to the ‘*d*’-like character of the valence charge density for the hypothetical isostructural face-center-cubic (FCC) → FCC transition in Cs is depicted in Fig. 2b [32].

**Figure 3.**
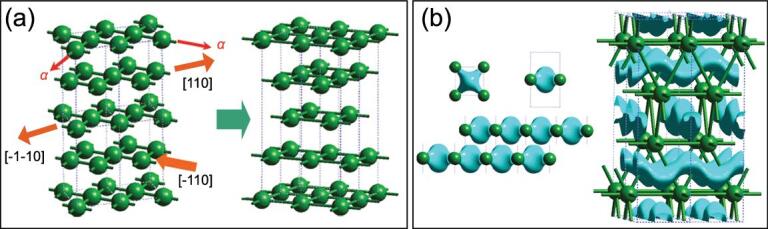
(a) The transformation from the FCC to the body-center-tetragonal (BCT) Cs-IV structure from the sliding of the 2D Cs planes and (b) the formation of wave-like charge density (electrides) from the overlap of the Cs *d* orbitals between two adjacent layers.

Later, re-examination of more refined diffraction patterns of high pressure Cs obtained using synchrotron X-rays showed the structural transitions are much more complicated [33]. Instead of an isostructural FCC transition, a new and complicated modulated orthorhombic layered structure with 84 atoms per unit cell (Cs-III, *C*222_1_) was found at 4.2 GPa [33]. The stability field of Cs-III was very narrow under ambient temperature, between 4.2 and 4.3 GPa, and disappeared upon cooling. Further compression led to a simple tetragonal structure (Cs-IV, *I*4_1_/*amd*) [34], a puckered layer structure (Cs-V, *Cmca*) at 12 GPa [35] and eventually the anticipated hexagonal close-pack structure (Cs-VI) appeared at 70 GPa [35]. This sequence of structural transformation has been analysed previously [29,36]. It was shown that the *s* → *d* transition (hybridization) led to the participation of the Cs 5*d* orbitals in the chemical bonding. The result of multi-centre overlaps between Cs 5*d* orbitals led to the formation of ‘electrides’, localized charge density at centres of the 2D square lattice network formed by Cs. The non-atom maximum electron density in dense polymorphs of Cs is perhaps the first example of electrides at high pressure. The electride is a well-known entity in chemistry [37]. The localization of electrons in the interstitial can be explained as the sharing of electrons among multiple atomic centres. Multi-centre bonding is energetically favourable in a (electron-deficient) system with more available orbitals than the number of electrons needed to completely fill them. In Cs, instead of one 6*s* orbital per atom, at high pressure the electron can access five *d* orbitals. It is interesting to note that in the 2D multi-centre bonding network, no electron density is observed between nearest-neighbour Cs atoms. The general notion of a chemical bond between a closest pair of atoms is not followed. Instead, a ‘formal’ bond occurs between second-nearest neighbour atoms. The adoption of in-plane Cs *d*-bonds weakens the 3D network of the precursor, transforming it into a structure consisting of layers of 2D square lattices that can ‘slide’ against each other forming Cs-III [29,36] and eventually forming Cs-IV (Fig. 3a).

An intricate feature of the chemical bonding in Cs-IV, revealed in the electron structure calculation by von Schnering and Nesper [38], is a novel wave-like pattern of electron density propagated between planes of the Cs atoms. The sinusoidal electron density distribution can be rationalized visually by sliding the 2D square lattice layers along two different crystallographic directions (Fig. 3b) [29,36]. Recently, there has been a renewed interest in the investigation of electrides in high pressure crystals due partly to their ubiquitousness and partly to the novel electronic structure that may be relevant to superconductivity [39–41].

**Figure 4.**
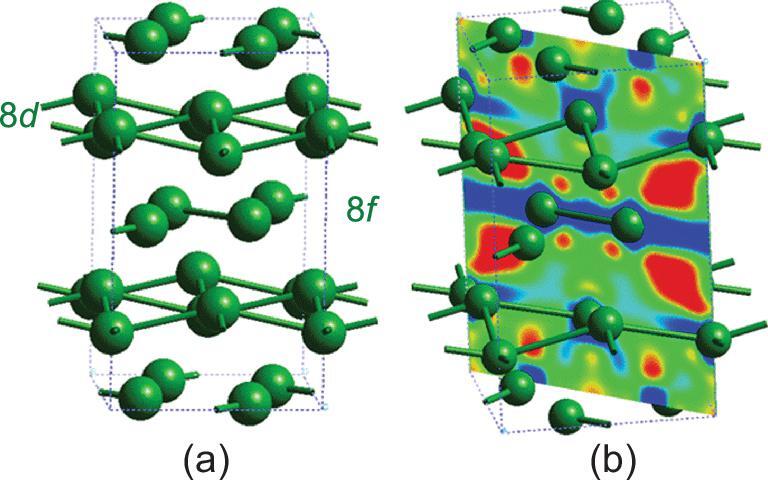
The (a) Cs-V structure and (b) electron density difference map relative to Cs atoms showing the electron transfer from the planar to the puckered layer.

Cs-IV transforms to Cs-V at 12 GPa (Fig. 4a) [42]. Cs-V has an orthorhombic structure consisting of alternate puckered and planar 2D layers of Cs atoms. An important feature of this structure is that this is the first Cs polymorph composed of two crystallographically distinct Cs sites. This observation implies that the two Cs atoms are also chemically different. The flat layer is constructed from Cs at Wyckoff 8*f* positions, and the puckered layer is constructed from Cs at the 8*d* sites. The calculated charge difference between Cs-V and atomic Cs illustrated in Fig. 4b [29,36] shows unambiguous charge transfer between the two sub-layers: depletion of electron density (blue region) is found in the flat layer and charge accumulation in the puckered layer (Fig. 4b). Chemical intuition would suggest that there will be no significant structural distortion in the electron-deficient 2D flat layer as electrons are removed. In contrast, the transfer of the electrons to the adjacent flat layer (electron-rich) will lead to a structural distortion. Charge transfer often suggests a tendency to open an energy gap in order to stabilize the overall structure. Although the transition to an insulator was not observed in Cs, pressure-induced metal → insulator transitions were indeed later found in elemental Li [43] and Na [44] under extreme compression.

**Figure 5.**
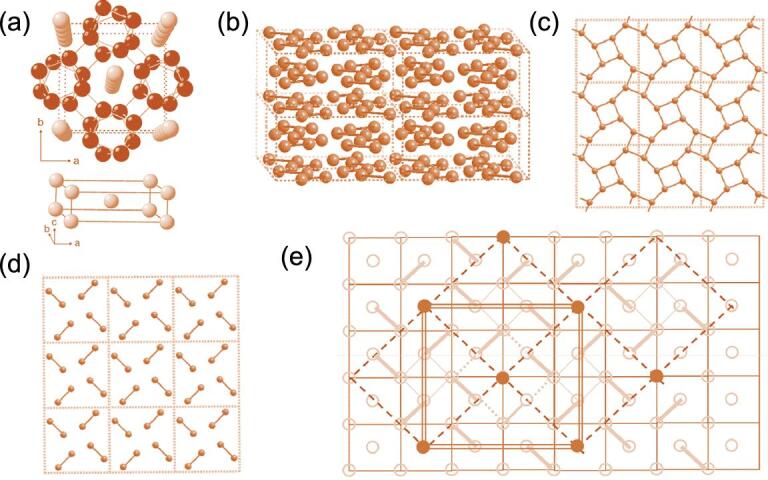
(a, b) Structure of the two sub-lattices of incommensurate Rb-IV; and (c–e) schematic representation of the sequence leading to the formation of the host–guest structure from the 2D square net derived from the precursor FCC structure.

The successive structural transformations in similar Group I Li and Rb elemental solids can be explained in the same manner. These transformations have already been discussed elsewhere [29]; here we only illustrate how the simple concept proposal for Cs can be applied to the elucidation of the guest–host structure in Rb-IV. When compressed from ambient pressure, Rb followed a transformation sequence similar to Cs [45–47]. A modulated structure (Rb-III) [48] was formed from the FCC phase (Rb-II) at 13 GPa [48]. Instead of transforming to the *I*4_1_/*amd* Cs-IV structure (Rb-V), a novel incommensurate guest–host structure was found at 17 GPa (Rb-IV) [47,48]. This phase was stable from 17 to 20 GPa and then eventually transformed to the Cs-IV-like *I*41/*amd*. In Rb-III, the Rb atoms forming the host framework were ordered and the atom positions followed the space group *I*4/*mcm*. On the other hand, the guest-atom positions conformed to the space group *I*4/*mmm* but were incommensurate with the host unit cell (Fig. 5a) [48]. To reconcile how this particular arrangement of Rb atoms arose from the precursor FCC structure, researchers first recognized the Rb-IV structure could be described as the stacking of alternate identical 2D square lattice layers of Rb atoms, rotated slightly relative to each other (Fig. 5b) along the *c*-axes [29]. As with Cs, Rb underwent an *s* → *d* hybridization in the post-FCC (Rb-II) phase forming 2D square lattices with electron density located in the middle of the squares (Fig. 5e). Schematically connecting the second nearest neighbour Rb atoms revealed a pattern, shown in Fig. 5d, where the square lattice motif is readily apparent. Connecting the second nearest neighbours in the square lattice left a single Rb atom surrounded by an 8-member ring. Figure 5c clearly reveals the guest–host framework in which guest atoms are situated in the channels (hole) perpendicular to the 8-member rings formed by Rb in the 2D planes.

**Figure 6.**
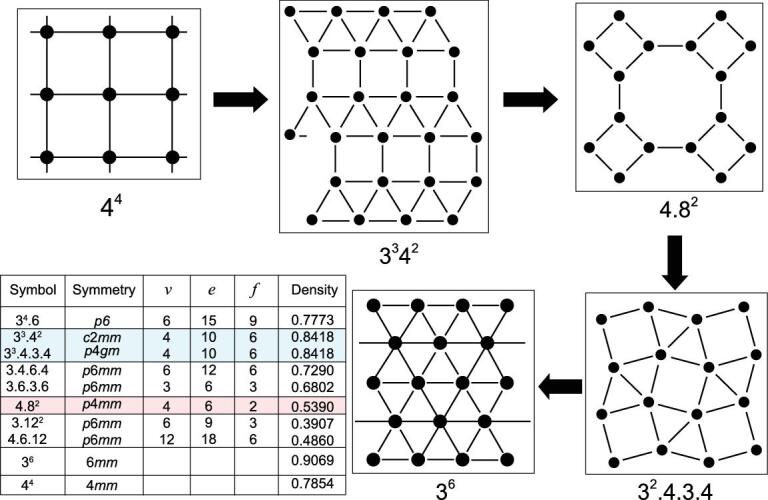
2D-Archimedean tiling of planar arrays of atoms with progressively higher packing density (see the inserted table).

The structural transformation of elemental alkali solids in the intermediate pressure range can be understood with the proposal of the formation of 2D square lattice layers. Remarkably, the structural types and sequence of successive structural transformations in Rb follows the same trend as the packing of hard spheres in two dimensions, i.e. the Archimedean tiling (Fig. 6) [49]. Even the unusual guest–host structures observed in Rb and Ba can be interpreted qualitatively with this model. Starting from the precursor cubic structure (FCC or body-center-cubic (BCC)), under pressure, structures of alkali and alkaline elements adopt the 2D square-net layer pattern after the *s* → *d* hybridization. The valence electrons are then ‘relocated’ (squeezed) into interstitial sites of the 2D lattice. As a result, the atomic size becomes smaller and the compacted atoms packed like hard spheres in the 2D plane [49].

**Figure 7.**
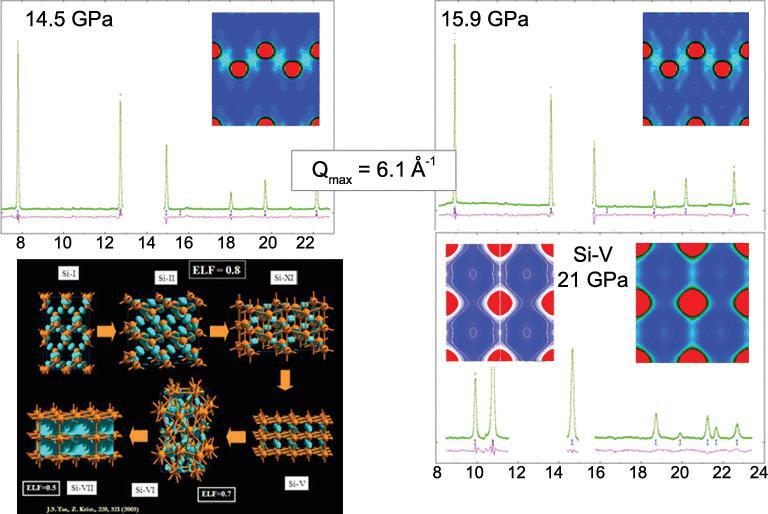
Calculated changes in the distribution of valence electrons in different high pressure polymorphs of Si. Low temperature diffraction patterns of Si and the corresponding electron density derived from maximum entropy method (MEM). The inset on the right at 21 GPa is the theoretical charge density (adapted from Tse *et al.* [50]).

**Figure 8.**
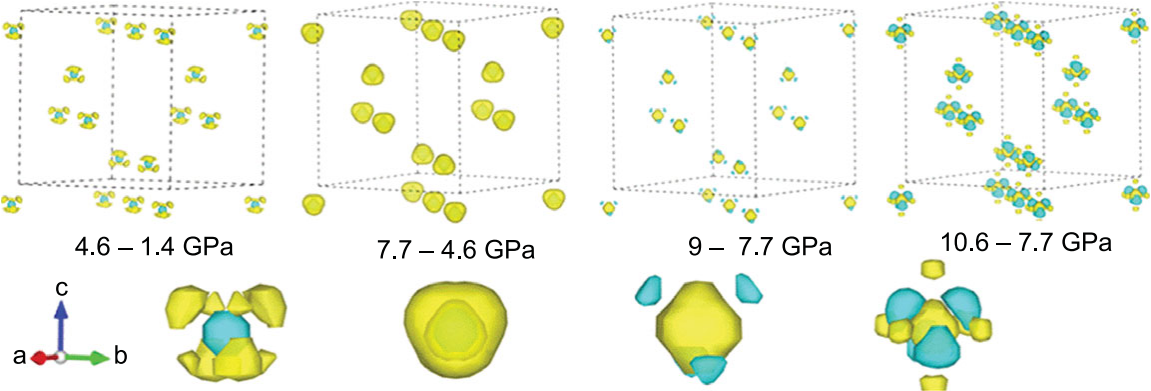
3D iso-surface maps of the difference in the electron distributions of crystalline Ge between structures at successive increasing pressure. Positive and negative charge differences are displayed as yellow and blue colours, respectively (adapted from Li *et al.* [52]).

The first experimental verification of orbital hybridization was demonstrated in the analysis of the electron density of compressed Si [50] from diffraction patterns using the maximum entropy method (MEM) [51]. Since valence electrons are mostly responsible for the intensities of low angle Bragg reflections, the MEM is an ideal method to extract valence electron density from truncated diffraction patterns obtained at high pressure. The results of the analysis of the diffraction patterns of compressed Si in a hydrostatic pressure transmission medium at 80 K and theoretical charge density calculations are shown in Fig. 7. The consistent agreements are striking. As anticipated, both theoretical and experimental electron densities show the ‘removal’ of electrons from the atomic sites into the empty void and the increasing participation of *d* orbitals in the chemical bond [29]. The *p–d* hybridization is more clearly demonstrated from a single crystal diffraction study of compressed Ge [52]. As shown in the charge density difference plot between successive pressures in Fig. 8, the *sp*^3^-like electron distribution weakens progressively with increasing pressure and concomitant increase in *d*-orbital character. The feature starts to become apparent at 7 GPa, below the phase transition to 11 GPa.

**Figure 9.**
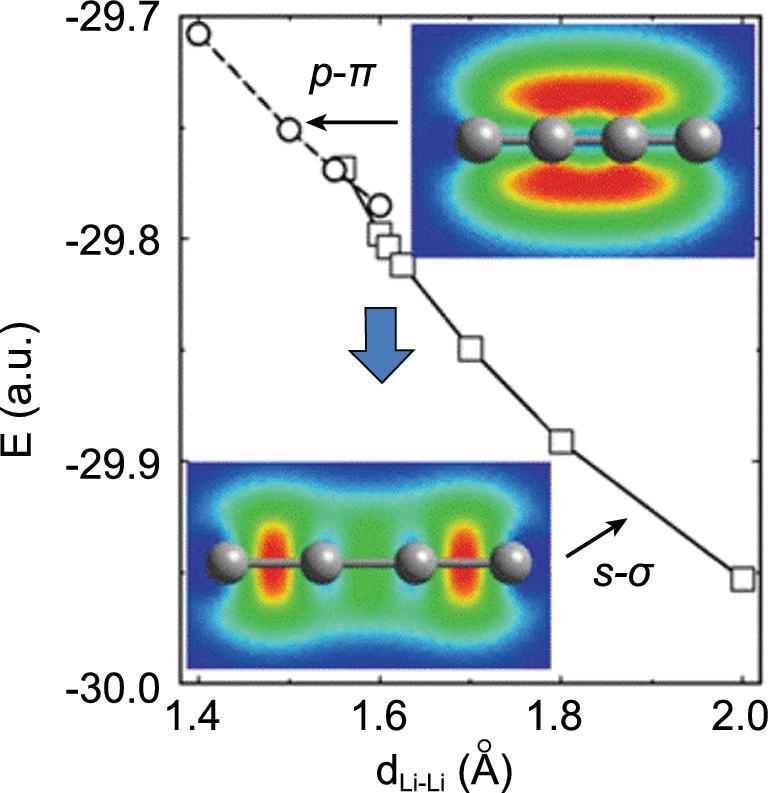
Total energy for a linear Li_4_ molecule as a function of Li-Li bond length (curve) and schematic representation of wave functions; note that the ground-state wave function for distances shorter than 1.5 Å consists entirely of Li–Li *p*_π_–*p*_π_ interactions (from Rousseau *et al.* [54]).

Research also suggests a redistribution of electron density may occur in high pressure Li [53,54]. It was observed from a band structure calculation that under high pressure the electrons are displaced from the atomic sites forming ‘dimer’ pairs. However, no electron density was found along the ‘bonding’ direction between two Li atoms forming the dimer [53]. Figure 9 shows the total energy and electron density of a hypothetical linear chain of Li atoms as a function of the Li–Li separation [54]. At a Li … Li distance shorter than 1.56 Å, the chemical bond changed from *sp*-σ to *p*_π_ - *p*_π_. Consequently, the electrons formerly in the σ bonds were relocated to the perpendicular π-bonds. The change in the bonding behaviour, i.e. formation of Li π-dimers with no electron density between the Li pairs, corresponds exactly as predicted from a band structure calculation [53]. Therefore, the localized chemical orbital picture is equivalent to the band description.

The discussions presented above suggest that when atoms are pressed against each other by external pressure, to alleviate the unfavourable Coulomb electron–electron repulsion, the valence electrons prefer to migrate into the empty spaces of the crystal to facilitate multi-centre bonding. This is similar to the effect of imposing a confinement potential on the atom. Under compression, the energies of the filled orbitals are pushed up allowing mixing with low-lying empty orbitals with higher angular momentum quantum numbers. In an orbital description, the spatially diverse directional hybrid orbitals enable redistribution of valence charge density (into the interstitial sites) and are responsible for the occurrence of a variety of the novel open-framework crystal structures. At extremely high pressure, when most valence electrons are already located in the empty space, the crystal structure of simple elements is mainly determined by the packing of the ions and therefore the close-packed structure re-emerges. For historical interest, it is noteworthy that Pauling had attempted to explain the structures of Cs-IV and Cs-V based on the assumption of cubic unit cells and came to the conclusion that both Cs-IV and Cs-V were composed of 122 and 162 atoms per unit cell, respectively [55].

## MIXING THE UNMIXABLE

**Figure 10.**
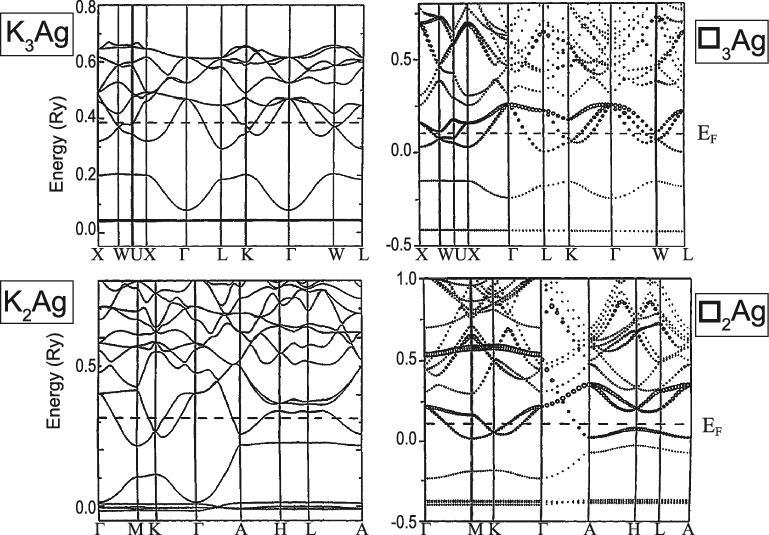
The valence band structures of K_2_Ag and K_3_Ag and of the corresponding hypothetical structure ■_2_Ag and ■_3_Ag with the K atoms removed (from Tse *et al.* [59]).

**Figure 11.**
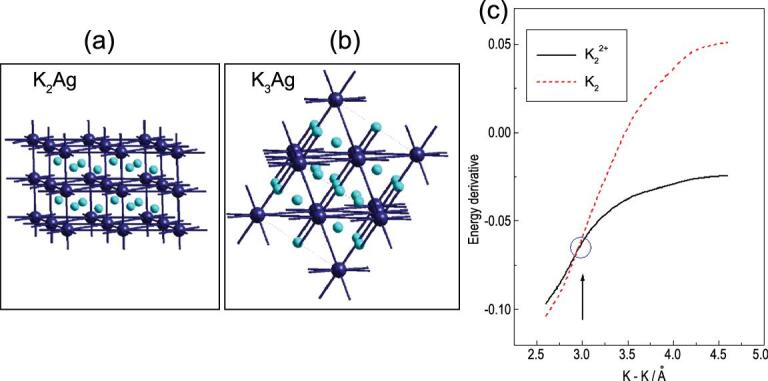
(a) The structure of K_2_Ag, and (b) K_3_Ag showing the graphitic and cubic Ag-network, and (c) the comparison of the potential energy curves for K_2_ and K_2_^2+^.

In a binary system, what happens when the second component (element) in the solid can accept the interstitial’s electrons? At ambient pressure, this kind of electron transfer is common between electropositive and electronegative elements. Perhaps the most familiar examples are the halides of alkali metals and Zintl compounds between main group elements [56,57]. Time-honoured empirical Miedema and Hume-Rothery rules govern the formation of a binary intermetallic alloy. The former states that the higher the electronegativity difference between two elements, the greater the heat of formation and hence, favourable alloy formation. The latter states that if the atomic size of two metals differs by more than 15% they will not form substitutional solid solutions [58]. An example is the K–Ag system [59]. Under normal pressure, due to the large difference in the atomic radii, K (2.43 Å) and Ag (1.65 Å) are immiscible and do not form alloys. It was a great surprise that when a mixture of the elements was compressed to a few gigapascals, crystalline alloys with stoichiometry K_2_Ag (4.1 GPa) with a hexagonal graphite-like layered structure and K_3_Ag (6.4 GPa) with a FCC structure were identified [20]. It was speculated the pressure required for the *s* → *d* transition in K was substantially reduced from 30 GPa in the pure element in the presence of Ag atoms. Since the K 3*d* orbitals are more compact than the K 4*s*, the hybridization reduced the size of the K atom making it more comparable to Ag and promoted alloy formation. This interpretation was later found to be incorrect [59]. Comparing the band structures of the alloys and the corresponding hypothetical Ag frameworks with K removed showed the K atoms transferred almost all the 4*s* electrons to the vacant Ag 5*p* orbitals; the K ions only acted as spectators (Fig. 10) in both K_2_Ag and K_3_Ag [59]. The Ag frameworks in K_2_Ag and K_3_Ag were constructed from overlaps of the Ag *sp* hybrid orbitals. A simple picture to describe the alloy structures has emerged. The ground-state electron configuration of neutral Ag is 4*d*^10^5*s*^1^. In K_2_Ag, two electrons, one from each K, were formally transferred to an Ag. The effective electron configuration of the resulting Ag atom was 4*d*^10^5*s*^2^5*p*^1^. The Ag valence orbitals then hybridized forming three *sp*^2^ hybrids. Chemical bonding between the *sp*^2^ hybrids formed a 2D honeycomb network of graphene-like layers stacked along the crystallographic *c*-axes, separated by an alternative layer of K cations (Fig. 11). From the same reasoning, in K_3_Ag, each Ag can acquire three electrons from the K atoms and the effective electron configuration of Ag becomes 4*d*^10^5*s*^2^5*p*^2^. Once again the Ag valence *s* and *p* orbitals rehybridized and bonding between the *sp*^3^ hybrids led to the FCC diamond structure. This interpretation infers the Ag atoms should have existed in −2 and −3 anionic states in K_2_Ag and K_3_Ag, respectively. This suggestion may be confirmed from the analysis of the electron densities extracted from the corresponding diffraction patterns using the MEM method described above. The relative ease of charge transfer from K to Ag under mild compression can be attributed to the large electronegativity difference (χ_K_ = 0.82 *vs.* χ_Ag_ = 1.93) of the neutral atoms at ambient pressure. Substantial electron transfer from K is reflected in the short K … K separation in the 2D layer of 3.13 Å, which is much shorter than the K–K distance of 3.82 Å in metallic K at the same pressure [20]. Previously, the very short K–K distance led to the proposal on the formation of K–K covalent bonds. This is again shown to be incorrect. Calculations of the total energy of the neutral K_2_ dimer and the dimer formed from K cations as a function of interatomic distance show the K–K interaction becomes less favourable compared to K^+^ ... K^+^ when the atom separation is shorter than 3 Å (Fig. 11c). Again, at high pressure it is energetically favourable to pack metal cations with smaller sizes than the neutral atoms. This analysis reinforces the concept that the valence *s* electrons are migrated from the diffuse *s* orbital to more tightly bound *d* orbitals as introduced above used to elucidate the high pressure structures of Cs and Rb (see below). After the *s*–*d* hybridization in Cs and Rb, the valence electrons were relocated to the interstitial sites. The high pressure structures followed the pattern of 2D close-packing of the cations (Archimedean tiling) [49].

**Figure 12.**
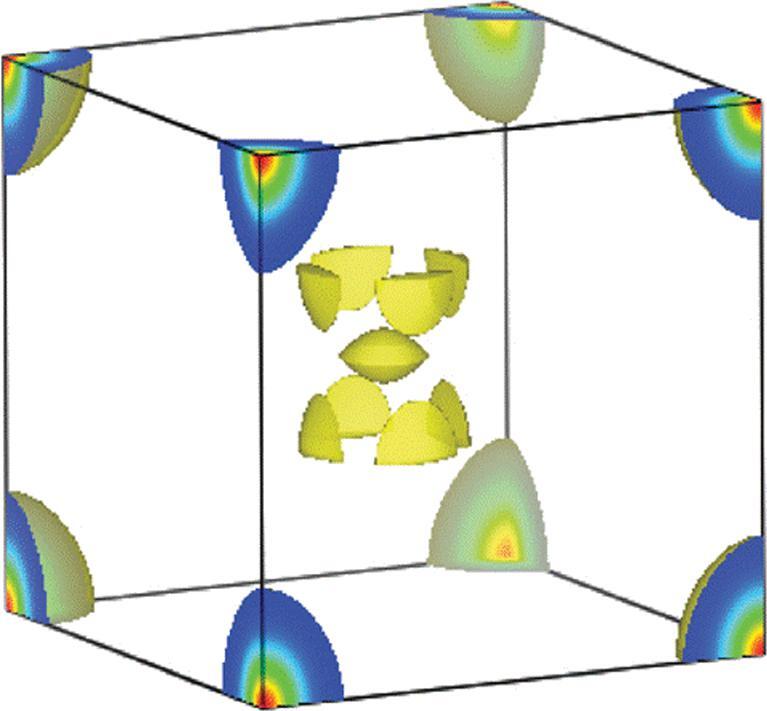
Three-dimensional electron density probability as obtained from analysis of X-ray diffraction data on a Li–Cs sample at 8 GPa and 298 K by the MEM (from Desgreniers *et al.* [21]).

A more striking example of mixing of the un-mixable is the formation of LiCs intermetallics [21]. Under normal conditions, Li and Cs are not miscible due to the large mismatch of their sizes. A theoretical structure investigation has predicted that stoichiometric binary Li–Cs alloys can only be formed at high pressures (>50 GPa) [60]. However, X-ray diffraction experiments provide evidence on the formation of new crystalline alloy(s) when a mixture of Li and Cs is compressed, even as low as 0.1 GPa [21]. This is the first observation of binary alloys between Group I elements. Experiments showed a non-stoichiometric Li_0.7_Cs alloy was formed at 1.6 GPa and remained stable at least up to 10 GPa [21]. The alloy structure was solved by the MEM method [51]. It had a simple cubic crystal structure and the Li and Cs positions were ordered. An important feature of the crystal structure is that the valence charge density derived from MEM analysis showed charge transfer from Cs 6*s* to the Li 2*p* orbital (Fig. 12). Recall that in the first row elements of low atomic number, the 2*p* orbitals have no radial node and the 2*s*, 2*p* energies are almost degenerated; yet charge transfer between electropositive alkali atoms in the same group was not anticipated. However, the electronegativity difference between Li and Cs in the Pauling scale of 0.2 is fairly large. The electron transfer helps to reduce the size of Cs (Cs^δ+^) and concomitantly increase that of Li (Li^δ−^). The mismatch in the atomic sizes becomes smaller leading to more efficient packing. The smaller Cs size is confirmed by the short interatomic distance of 3.858 Å as compared to 4.23 Å corresponding to pure metal (Cs-II) at similar pressure. The substantially shorter Cs–Cs separation in the alloy compared to the bulk metal is reminiscent of the K–K distance observed in K_2_Ag. Both observations are consistent with the interpretation that K and Cs are in the cationic state. At high pressure, the dispersion of electrons into the empty space makes compression of the cations energetically more beneficial.

It is significant that the experimental LiCs structure was not predicted by theoretical total energy calculations [60]. The non-stoichiometric structure makes theoretical prediction more difficult. It is pertinent to note that almost all structure prediction methods are designed to explore the global energy minimum structures. It is not uncommon that metastable structures are observed in experiments as the system may choose to take the lowest activation energy path in a structural transformation leading to a meta-stable structure.

**Figure 13.**
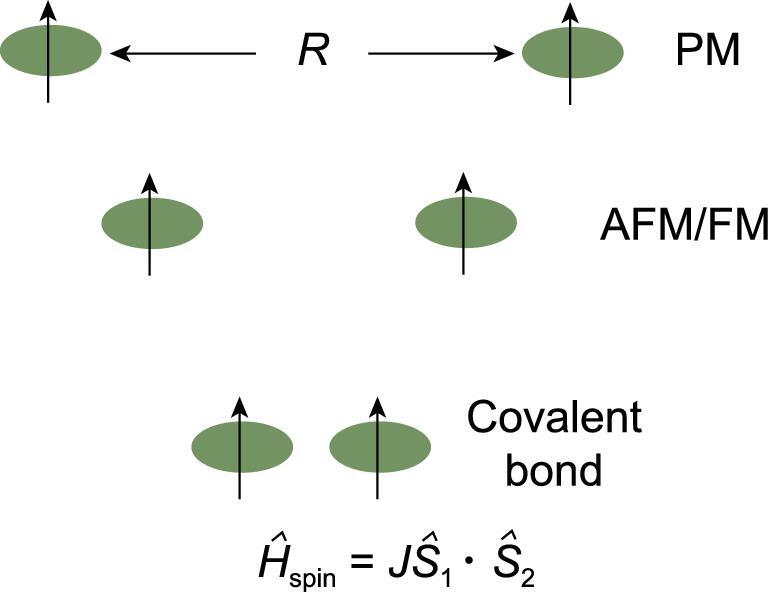
Schematic energy level diagram for the interaction of two electron spins as a function of their separation showing the formation of paramagnetic (PM), ferro-(FM) and anto-ferro-(AFM) magnetic states and the eventual formation of spin pair covalent bond. The green shades represent the atoms and not the spin orbital.

**Figure 14.**
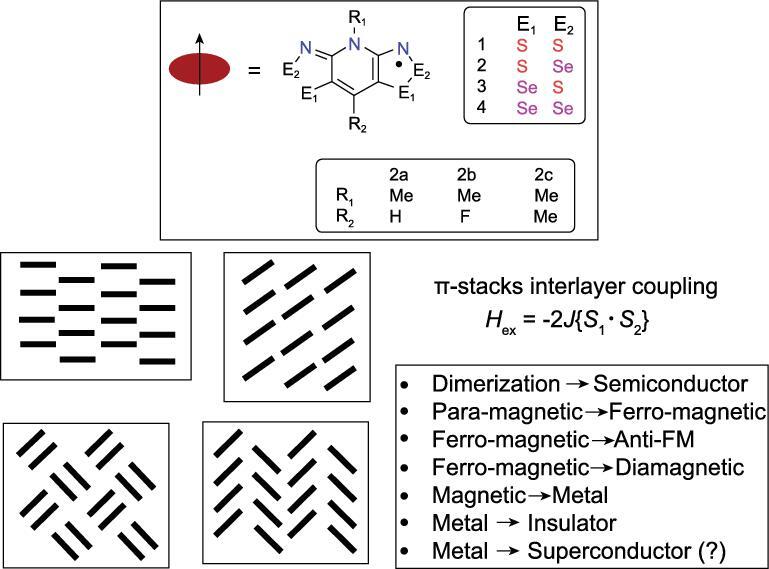
Structure of a neutral radical molecular building block. Structures of different molecular sizes can be created with different substituents E_1_, E_2_, R_1_ and R_2_. The morphology and the magnetic state of the crystal can be manipulated using building blocks with different sizes.

**Box 1.** A comparison of the order of magnitude estimates of the mechanical work by external pressure with typical bond strengths.

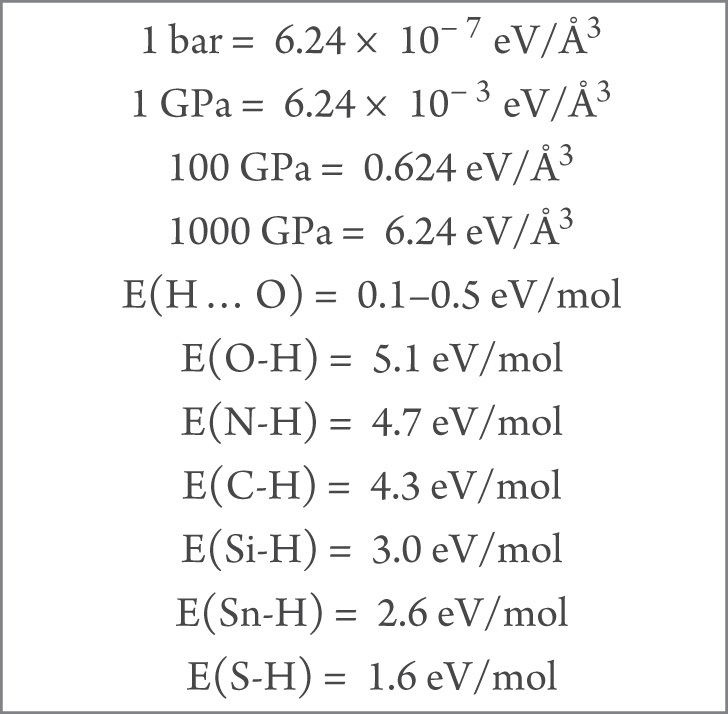

## THE ENERGETIC FACTOR

When a material is compressed, the mechanical (*PV*) work may help to overcome the energy barrier associated with the transformation into another crystalline form. If the external force is very large, it may alter the chemical bonds as well. Based on equations of the states of several molecular systems, rough estimates of the energetics of *PV* work by external pressure are compared in Box 1 [29]. At a few gigapascals, the pressure only slightly perturbs the crystal structure and does not affect the chemical bonds. However, even at the very low pressure regime, the small structural modifications can affect the material’s electrical and magnetic properties significantly, particularly for molecular complexes.

**Figure 15.**
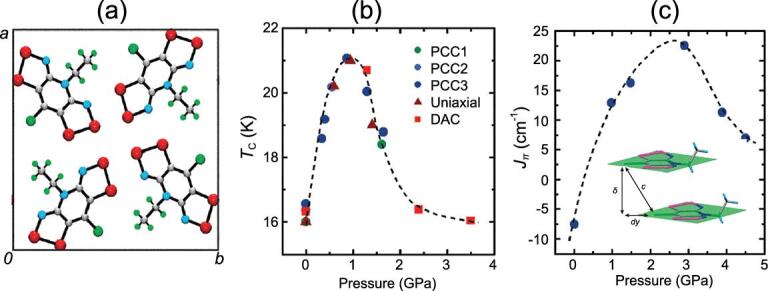
(a) Crystal structure of the neutral radical with E_1_ = E_2_ = Se and R_1_ = Et, R_2_ = Cl. (b) The Curie temperature (*T*_c_) measured under hydrostatic (PCC), uniaxial, and in a diamond anvil cell. (c) Calculated Heisenberg coupling parameter *J*_π_ as a function of crystal structure geometry at different pressures.

To illustrate this point, Fig. 13 shows the interactions between two identical open-shell species, each with an electron in a singly occupied orbital (such as two H atoms), as a function of the separation. At large separation, the two moieties are independent of each other, their spins are randomly oriented and the magnetic (spin) state of the total system is paramagnetic. As the two species are brought closer, the electron spins start to interact. In the Heisenberg model, depending on the exchange parameter (*J*), the ground magnetic (spin) state (arrangement of the electron spins) can be diamagnetic (spin-paired), ferromagnetic (FM) or anti-ferromagnetic (AFM). Reducing the separation even further leads to the formation of a genuine two-electron spin-paired covalent bond. A molecular radical, shown in Fig. 14, is a convenient model for the study of pressure-induced metal → insulator transition. A model system can be realized using stable molecular radicals as the building blocks of the crystal structures. One class of such molecular radicals is the bisthia/selenazolyl radical and its chemical variants (Fig. 14) [61–76]. Manipulation of the substituent groups R (alkyl group and halogen) and E (chalcogen element, S and Se) (Fig. 14) can lead to different stacking of the molecular radicals in the solid state and different magnetic ground states (Fig. 14). It is possible to control the magnetic property of these molecular solids by changing the separation between the radical molecules. The initial strategy was to make use of chemical pressure through the synthesis of similar compounds with R groups of different sizes. This is a laborious procedure and often not very effective. A better strategy is to apply physical pressure. The first high pressure experiment in a diamond anvil cell was performed on bis-selenazolyl with R_1_ = Et, R_2_ = Cl (Fig. 15a) [75]. The results were remarkable. At ambient pressure, a paramagnetic → ferromagnetic transition was found at low temperature. The transition temperature (Curie temperature) increased with applied pressure and reached 21 K at 0.9 GPa (Fig. 15b). This is the highest Curie temperature ever reported for a molecular magnet. X-ray diffraction showed there was no change in the internal molecular structure under very mild pressure. Instead, the molecular radicals slipped against each other (*dy*, the relative displacement between two parallel molecules in close contact, in Fig. 15c) and concomitantly brought the molecules closer. The intermolecular interaction between neighbouring radicals along the π-stacks changed the spin-exchange coupling parameter *J*_π_. Initially *J*_π_ increased with pressure due to the reduction of overlap between adjacent singly occupied molecular orbitals. This led to stronger ferromagnetic interaction and higher Curie temperature. Upon further compression, the slippage of the π-stacks moved past minimum (orthogonal) overlap and *J*_π_ began to decrease and so did the Curie temperature.

**Figure 16.**
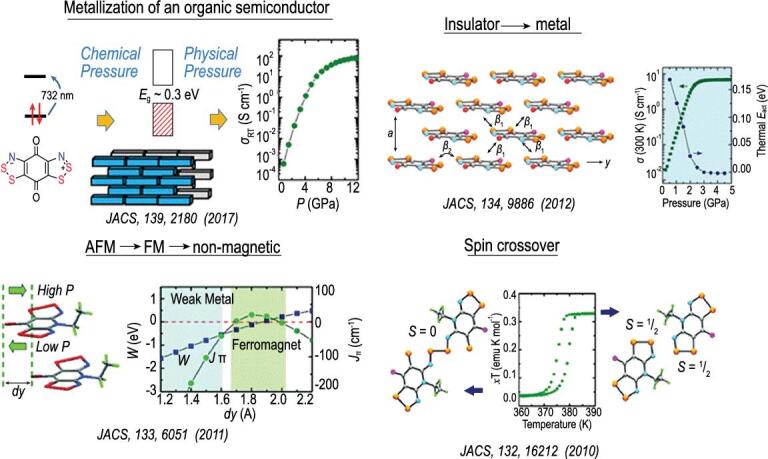
A summary of the magnetic properties of different neutral radical solids.

The example presented above shows that change in the van der Waals interaction under small external pressure can significantly affect the magnetic property of a molecular crystal. The usual behaviours of an ordinary magnetic system, i.e. paramagnetic → FM ordering, paramagnetic → AFM ordering, FM → AFM and insulator → metal transition have been demonstrated by compressing this class of molecular crystals with selected variants of bisthia/selenazolyl radicals (Fig. 16) [61–76]. In any case, the molecular structure was found to be significantly distorted. These molecular crystals are air stable and can be synthesized with high purity. Doping is not necessary to alter the electronic and magnetic properties. The crystal structure can be determined precisely *in situ* under high pressure and low temperature. The electronic structure of the molecular radical is inherently strongly correlated. This class of crystals has served as an excellent model for the investigation of magnetic and metal-to-insulator transitions and correlated effects [67].

## STRUCTURAL TRENDS IN HIGH PRESSURE POLYHYDRIDES

Interest in the investigation of the structures and properties of polyhydrides at high pressure originated from the proposition that polyhydrides inherently have a higher concentration of H than molecular hydrogen; therefore, less pressure may be required to compress them into the metallic state. Moreover, at the desirable electron density, superconductivity may occur [77]. Recent research on this topic has benefited substantially from the availability of structure prediction techniques based on First Principles electronic structure calculations [78–80] in late 2000. This critical theoretical development offers a sound and logical way to explore stable high pressure structures otherwise not amenable to experiment. Since then, many studies on binary and ternary polyhydride systems with different elements have been reported. Most work reports the thermodynamically stable (global minimum) structures at a given pressure but there are few in-depth analyses of the evolution of the structures as a function of the pressure or hydrogen concentration. As shown in Box 1, the bond strengths of common main group elements with H are within the order of a few electronvolts; therefore, bond breaking is possible if the external pressure is in the megabar range. For example, it was predicted that SiH_4_ transforms to a superconducting phase around 60 GPa. Among the many theoretical studies on polyhydrides [23], the structures of strontium hydrides have been analysed in great detail. This research has helped to shed light on possible mechanism(s) for the occurrence of H^−^ at low pressure and the formation of H networks at high pressure with increasing H content [81].

**Figure 17.**
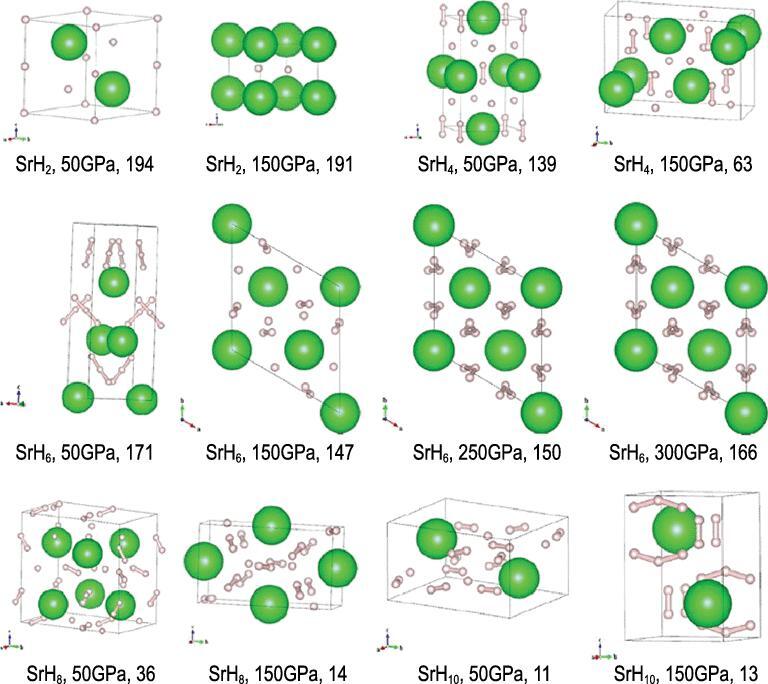
Predicted stable structures of SrH_2*n*_ (*n* = 1–5) at different pressures (from Wang *et al.* [81]).

**Figure 18.**
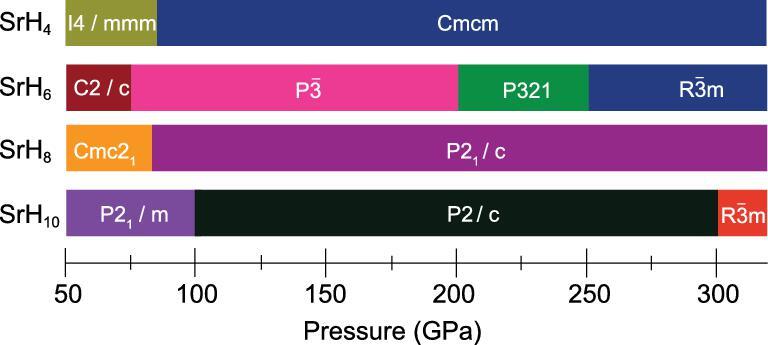
Predicted phase transition sequences of strontium polyhydrides with increasing pressure (adapted from Wang *et al.* [81]).

The global minimum structures of SrH_2*n*_ (*n* = 1–5) in the pressure range 50–300 GPa predicted by the particle swarm optimization method are shown in Fig. 17 [81,82]. Examination of the high pressure polymorphs reveals a trend in the structural motifs. At low H concentration, such as in SrH_2_, up to 60 GPa, the H atoms remain as monatomic anions. In SrH_4_, an equal mixture of monatomic H and H_2_ dimers is found in structures up to 150 GPa. The morphologies of the H structural units are more diverse in SrH_6_. At 50 and 150 GPa, the polymorphs are formed from monatomic H and transform to bent H_3_ units at 200 GPa. Upon further compression to 250 GPa, the H_3_ units are linked forming spiral chains. In SrH_8_, once again the H and H_2_ moieties are found in the 50 GPa structure. At 150 GPa, a mixture of H_2_ and bent H_3_ units emerge. The structural evolution of the high pressure SrH_10_ polymorphs follows a similar pattern. The most stable structure at 50 GPa again consists of H^−^ and H_2_ units but transformed to H_2_ and bent H_3_ moieties at 150 GPa. Interestingly, at 300 GPa, the structure is composed of puckered hexagonal honeycomb layers of H atoms. The stability fields of SrH_2*n*_ structures with different H stoichiometries are shown in Fig. 18.

The structural trend is not unexpected from a chemical viewpoint. At low H concentration, e.g. SrH_2_, one assumes two electrons from the electropositive Sr are donated to H_2_, occupying the anti-bonding H–H orbital and thus dissociating the molecule forming ionic Sr^2+ …^ H^−^. At higher H concentration, assuming the electrons are shared among all the H_2_, there will not be enough electrons to break the H–H bonds. The system has two alternatives: (i) breaking selected H_2_ forming monatomic H and/or (ii) sharing the donated electrons between clusters of H atoms and maintaining some H–H bonds. At low pressure, mechanism (i) is energetically more favourable. Therefore, monatomic H and H_2_ species were often observed in the low pressure structures of SrH_2*n*_ (*n* = 2–5) and other main group hydrides. At higher pressures, H and H_2_ are pushed closer. To alleviate electron repulsion, it is preferable to form H_3_ units by sharing the electrons donated by Sr among three H atoms. In H_3_, the third electron is placed in the non-bonding orbital and therefore the system still retains some stability. Further compression leads to the linking of H_3_ units as spiral chains and puckered 2D honeycomb layers.

**Figure 19.**
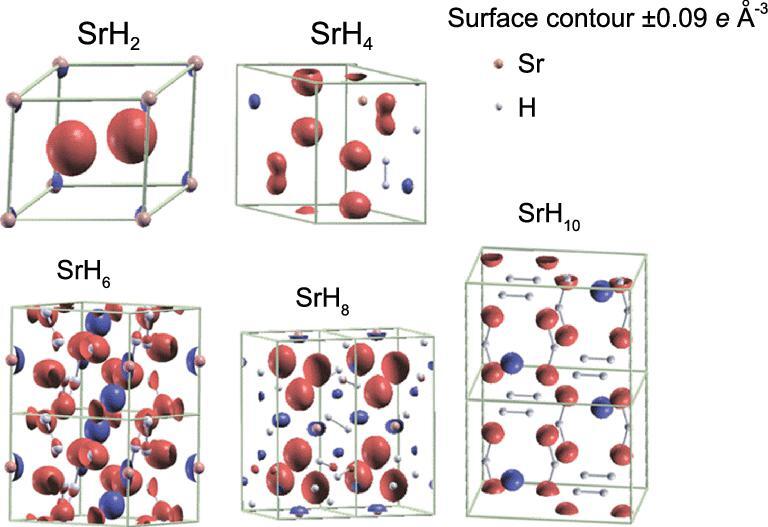
The iso-surface of the charge density difference for SrH_2_ (*P*6/*mmm*), SrH_4_ (*Cmcm*), SrH_6_ (*P*-3), SrH_8_ (*P*21/c) and SrH_10_ (*P*2/*c*) at 150 GPa. The charge depletion and accumulation are shown in blue and red, respectively (from Wang *et al.* [81]).

The qualitative description presented here assumes the formal transfer of two valence electrons from the Sr atom. This assumption is supported by the examination of the charge density differences, Δρ = ρ (SrH_2*n*_) − [ρ (Sr■_2_) + ρ(■H_2*n*_)], where ■ indicates vacant Sr or H sites in the corresponding SrH_2*n*_ crystal structure. A comparison of the charge density differences for the SrH*_n_* (*n* = 1–10) at 150 GPa is shown in Fig. 19. Indeed, significant electron transfer from Sr to the H species is observed in all structures.

The driving force for the electron transfer is again related to the electronegativity difference between Sr and H_2_. Mulliken defined the electronegativity of a species as the arithmetic mean of the ionization potential and electron affinity. These quantities can be computed from density functional theory as the first and second derivatives of the variation of total energy with respect to the number of electrons [83]. Using an almost completed atomic basis set for H, the electronegativity of a H_2_ molecule is calculated to be 1.71 in the Pauling scale [81]. This value is remarkably close to the electronegativity of Group XIII and XIV elements (1.61–2.33) that are known to form Zintl intermetallic compounds with alkaline metals [84]. The electronegativity of Sr in the Pauling scale is 0.95. The large electronegativity difference (Δχ = 0.76) between Sr and H_2_ suggests ionic bonding in the SrH_2_ polymorphs is feasible. It is noteworthy that when the H concentration far exceeds the accessible electrons donated by the metal atoms, only a small fraction of the electrons is acquired on individual H_2_. In this case, no significant change occurs in molecular H–H bonds. Thus, at low pressure, the H_2_ molecular species prevail, and the crystal structure consists of mainly non-dissociated H_2_ molecules. This simple description has been successfully applied to understanding the structural trend of calcium polyhydrides [85]. A cautionary note on the application of the electronegativity concept at high pressure is that, in principle, electronegativity is only defined for elements at ambient pressure. Thus, the model may only apply to polyhydrides composed of metals with low first ionization energy orbitals and should not be used indiscriminately. For example, hydrogen-rich Group 17 chlorine (H*_n_*Cl, *n* = 2–7) compounds behave quite differently from the metal hydrides [86].

## SUPERCONDUCTIVITY IN DENSE POLYHYDRIDES

As mentioned above, the impetus of the study of the crystal and electronic structures of polyhydrides at high pressure is to search for potential superconductors with high critical superconducting temperatures (*T*_c_) [77]. The current status on this topic has been reviewed in the work of Wang *et al*. [87], Shamp and Zurek, and Bi *et al*. [88].

**Figure 20.**
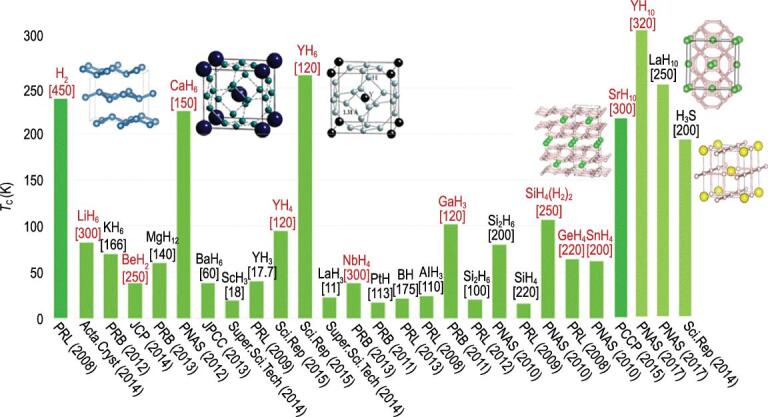
Predicted superconducting temperature of selected polyhydrides (adapted from Ma *et al*. (private communication)). Structures for the hydrides with high *T*_c_ are illustrated. The numbers in square brackets indicate the pressure in which the *T*_c_ was calculated.

The search for superconductivity in dense metallic polyhydrides was started before the availability of First Principles structure prediction methods. The first numerical calculation of the superconducting critical temperature of a hydride at high pressure using a modern First Principles electronic structure method [89,90] was on silane, SiH_4_ [91]. A high pressure structural model was obtained by combining finite-temperature constant-pressure molecular dynamic simulation and *ab initio* geometry optimization. An insulator → metal transition with indirect gap closure was found at 60 GPa. Electron–phonon coupling parameter (λ), an indicator of the strength of potential superconductivity, of the high pressure phase based on the Bardeen–Cooper–Schrieffer (BCS) phonon-mediated theory [89,90] was calculated at 90 and 125 GPa using the linear response perturbation method [89]. Large electron–phonon couplings close to 0.9 were predicted. Employing the McMillan equation [92], *T*_c_ was estimated to be between 45 and 55 K. At the time, the result was remarkable as it was believed that the upper limit of the *T*_c_ attainable by the BCS mechanism was about 40 K [92]. Later, calculations on SnH_4_ predicted an even higher *T*_c_ of 80 K at 120 GPa [93]. Superconductivity in SiH_4_ was indeed observed in an experiment [94]. A *T*_c_ of 17 K at 120 GPa was reported. However, the nature of the superconducting phase is still under debate. One suggestion attributes the superconductivity to the formation of superconductive PtH  from the reaction of the Pt electrodes with decomposed SiH_4_ [95]. This speculation is not reasonable since the Pt electrodes were not touching, so if even PtH was superconducting [96] and the silane was not, no superconductivity would be detected. Moreover, further compression during the experiment to 150 GPa led to the formation of the theoretically predicted stoichiometric insulating SiH_4_ phase [94,97] with no detectable Si impurity in the diffraction pattern if the reaction with Pt had occurred. The numerical calculations and experiment confirm the proposal that superconductivity can be achieved by compressing ‘pre-densified’ hydrides [77]. More recently, a record-setting *T*_c_ of 200 K was reported on compressed H_2_S albeit at 200 GPa [8]. An ingenious synchrotron Mossbauer nuclear forward-scattering experiment using Sn foil as a probe has demonstrated the Meissner effect unequivocally [98]. A far-infrared experiment also confirmed that the energy of the superconducting gap is comparable to the prediction of BCS theory [99].

Initial calculations on SiH_4_ and SnH_4_ were followed by a plethora of theoretical reports on a variety of high pressure hydrides with elements in the periodic table, many showing *T*_c_ exceeding 100 K at pressures over 100 GPa [87,88]. A distinctively high *T*_c_ of 220–235 K at 150 GPa was predicted for CaH_6_ [85]. Unlike other hydrides studied before, CaH_6_ has a unique sodalite structure with H atoms forming the open clathrate framework with Ca atoms encaged in the cavities (Fig. 20). Subsequently, YH_6_ [100], YH_10_, LaH_6_ and LaH_10_ with clathrate-like structures [101,102] were predicted to be high performance superconductors with *T*_c_ close to room temperature at a pressure ∼200 GPa [8]. A recent experiment confirms LaH_10_ has a *T*_c_ of 260 K between 180 and 200 GPa [9]. A summary of selected high *T*_c_ polyhydrides is shown in Fig. 20. Two common structure motifs emerge for the high *T*_c_ polyhydrides, one with the H-clathrate structures and the other with puckered honeycomb layers of H atoms. Why do the clathrate H-atom framework and the 2D puckered honeycomb layered structures facilitate such high *T*_c_?

To understand the fundamental mechanism for the strong electron–phonon coupling in hydrogen-rich alloys, the Eliashberg equation [103] was solved within the Migdal–Eliashberg theory using the spectral functions (α*F*(ω)) obtained from electronic structure calculations [98,99]. The Eliashberg spectral function contains all the information concerning the electron–phonon coupling of individual vibration mode. It is defined as [90,104]:

(1) \begin{equation*} {\alpha}^2F\left(\omega \right)=\frac{1}{2\pi N\left({\epsilon}_F\right)}\sum_{\boldsymbol{q}\!\upsilon}\frac{\gamma_{\!\boldsymbol{q}\!\upsilon }}{\omega_{\boldsymbol{q}\!\upsilon }}\delta \left(\omega -{\omega}_{\boldsymbol{q}\!\upsilon}\right)\!, \end{equation*}

where *N* (ε_*F*_) is the electronic density of states at Fermi energy *ε_F_*. The phonon linewidth is

(2) \begin{eqnarray*} {\gamma}_{q\upsilon}&=&2\pi {\omega}_{\boldsymbol{q}\!\upsilon}\sum_{\mathit{kjj}^{\prime }}{\left|{g}_{k+{j}^{\prime}\!, \mathit{kj}}^{\boldsymbol{q}\!\upsilon}\right|}^2\delta \left({\epsilon}_{\mathit{kj}}-{\epsilon}_F\right) \nonumber\\ &&\times\,\delta\left({\epsilon}_{k+{q}_{j\prime }}-{\epsilon}_F\right)\!, \end{eqnarray*}

where the electron–phonon interaction matrix element is

(3) \begin{equation*} {g}_{k+{\boldsymbol{q}}\!_j\prime}^{\boldsymbol{q}\!\upsilon }=\left\langle k+{q}\!_{j\prime }\ \left|{\delta}^{\boldsymbol{q}\!\upsilon }V\right| \mathit{kj}\right\rangle\!,\end{equation*}

in which the electronic state *k_j_* is coupled with a phonon mode ω_***q****ν*_ and scattered to *k* + *q*_*j^′^*_.

**Figure 21.**
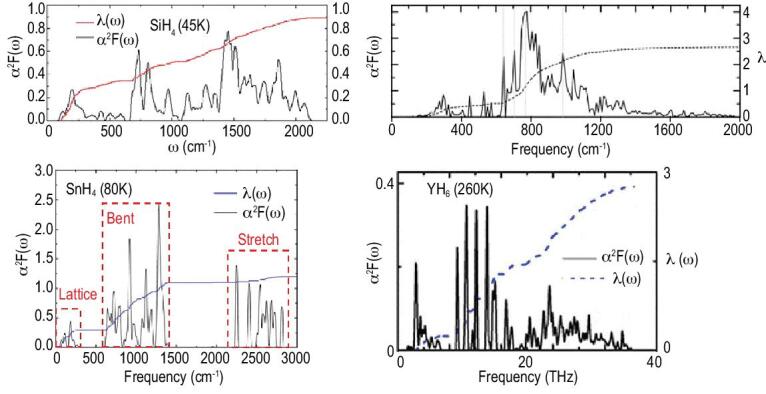
The Eliashberg electron–phonon spectral function (α^2^*F*(ω)) for SiH_4_, SnH_4_, CaH_2_ and YH_6_.

The Eliashberg spectral functions for four representative hydrides SiH_4_ [91], SnH_4_ [93], CaH_6_ [85] and YH_6_ [100] with increasing *T*_c_ of 45, 80, 230 and 260 K are compared in Fig. 21. In SnH_4_, molecular-like H_2_ is intercalated between two layers of Sn atoms [93]. The spectral function is clearly separated into three regions, translation, libration and H–H stretch. The integrated electron–phonon coupling constant λ(ω) shows the main contribution arises from the libration vibrations with smaller contribution from the translation modes. It is surprising that the high frequency (>2000 cm^−1^) H–H internal stretch does not contribute significantly. This contradicts the simplistic expectation that the higher the ‘mean’ frequency the larger the electron–phonon coupling [92]. In SiH_4_ the H_2_ bridges two Si atoms in a 2D layer. Now the stretch and libration vibrations are mixed and there is no longer a clear separation into two distinct vibration regions. However, in these two systems, the electron–phonon couplings are weak. For example, the overall electron–phonon coupling parameter is λ = 0.9 in SiH_4_ and is slightly lower than SnH_4_. On the other hand, the spectral functions for CaH_6_ and YH_6_ with the sodalite structures are distinctively different from SiH_4_ and SnH_4_. In the clathrate structure, the H atoms are connected forming an open framework structure and there are no more localized H–H vibrons. The integrated λ increases continuously with the vibrational frequency showing all phonons participate in the electron–phonon coupling giving very high λ, 2.69 in CaH_6_ [85] and 2.93 in YH_6_ [100].

**Figure 22.**
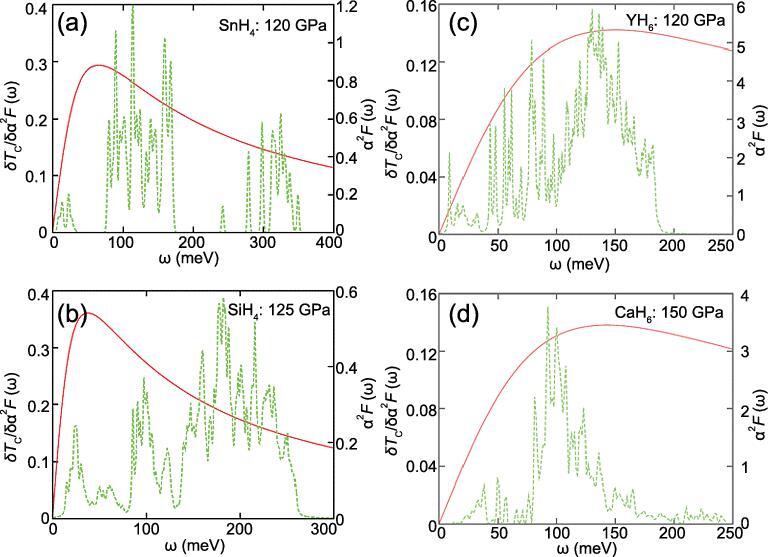
The functional derivative of the Eliashberg spectral function for SnH_4_ (a), SiH_4_ (b), YH_6_ (c) and CaH_6_ (d) (from Tanaka *et al.* [106]).

The functional derivative of the spectral function, δ*T*_c_/δα^2^*F*(ω) [105,106], enables the identification of the frequency regions where electron–phonon couplings are most effective. Results of the analysis of the same four hydrides are summarized in Fig. 22 [106]. The maximum in the δ*T*_c_/δα^2^*F*(ω) *vs.* ω plot is the optimum frequency (ω_opt_) for electron–phonon coupling for a given system. Deviation from this frequency will deteriorate the *T*_c_. The optimum frequencies for SiH_4_, SnH_4_, CaH_6_ and YH_6_ are 38, 65, 150 and 143 meV (1 meV = 8.0655 cm^−1^ = 2.4178 THz), respectively. It is known that the optimum frequency ω_opt_ ≈ 7 *k*_B_*T*_c_ [104], and the calculated ω_opt_ follows the trend of increasing *T*_c_. In SiH_4_ and SnH_4_ the optimum frequency peaked at or just above the translation vibration region. The results show the libration and stretch vibrations are not effective to enhance electron–phonon coupling. In comparison, the ω_opt_ of CaH_6_ and YH_6_ are close to the highest cutoff frequency of the respective vibrational spectra (Figs 21 and 22). In the latter two systems, the most efficient phonon modes for electron–phonon coupling are the libration modes. The absence of H–H vibrons in the clathrate structure suggests phonon modes are concentrated in the librations (i.e. high vibrational density of states in this region).

**Figure 23.**
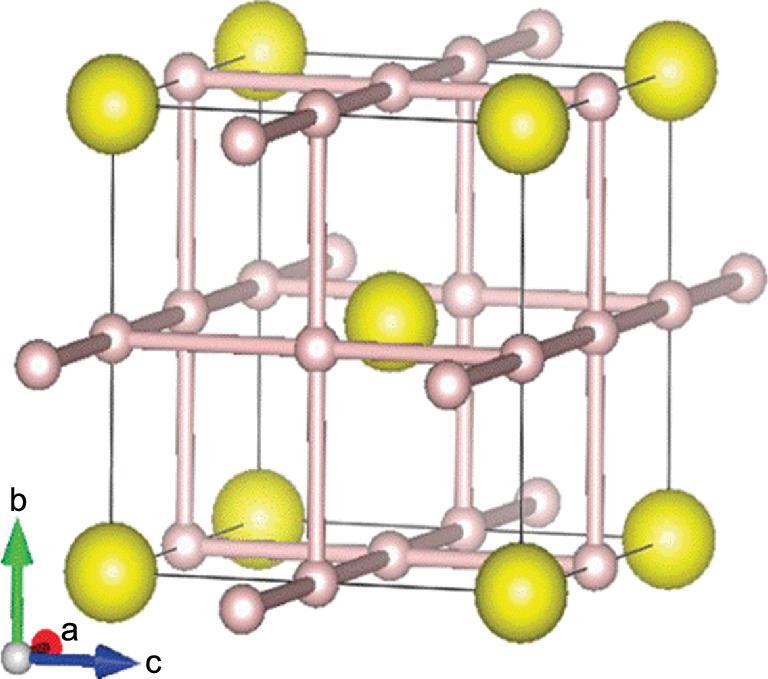
Crystal structure of predicted H_3_S showing the encapsulation of S inside the H-cage (from Tanaka *et al.* [106]).

It is commonly believed the superconducting phase of compressed H_2_S is H_3_S, the decomposed product of H_2_S under extreme pressure [8]. Apparently, both observed *T*_c_ and the *in situ* X-ray diffraction pattern [107] are in good agreement with the theoretically predicted H_3_S structure [108]. The H_3_S crystal structure is BCC with sulfur atoms located at the centre of a cubic box created by a 3D hydrogen network (Fig. 23). In a way, this structure is similar to the clathrate-like structures. The cutoff frequency of the vibrational spectrum of around 2000 cm^−1^ shows the absence of high frequency molecular-like vibrons. Analysis of the Eliashberg function of the theoretical H_3_S structure [109] has been performed and the optimum frequency obtained from the derivative of the Eliashberg function of ∼120 meV is comparable to that of CaH_6_ and YH_6_. A comparison of *T*_c_, ω_opt_ and ω_log_, and the H–H bond lengths is given in Table 1.

**Table 1.** Comparison of the *T*_c_, optimal frequency ω_opt_, the average phonon frequency ω_log_ and the H–H distance in the H network for the systems discussed here.

System	Pressure (GPa)	*T*_c_ (K)	*ω*_opt_ (meV)	*ω*_log_ (meV)	H-H distance (Å)
SiH_4_	125	53	38	79	–
SnH_4_	120	98	68	76	0.841
YH_6_	120	247	150	63	1.306
YH_10_	250	291	177	95	1.132
YH_10_	300	275	170	125	1.029
LaH_10_	300	231	144	123	1.076
CaH_6_	150	235	143	87	1.238
SrH_10_	300	259	159	66	0.997

There are two main contributions to a large electron–phonon coupling parameter. The area of the spectral α^2^*F*(ω) gives the total strength of electron–phonon coupling and the functional derivative indicates the most efficient phonon modes for coupling [104]. Since the vibrational profile is mainly determined by the structure (chemical bonding) of the crystal, analysis of the Eliashberg spectral function reveals that only structures with interconnected H networks (such as clathrates and 2D honeycomb H layers) with no H_2_ vibrons and the *T*_c_ for those systems are higher.

Can a polyhydride with *T*_c_ higher than room temperature be realized? So far, experiments support (see below) the BCS phonon-mediated superconductivity mechanism as valid in high pressure hydrides. Previous studies [14,109] have demonstrated a linear relationship of the superconducting gap 2Δ_0_/*k*_B_*T*_c_ with the logarithmic phonon frequency (ω_log_) [110] for superconductors obeying the BCS mechanism.

(4) \begin{equation*} {\omega}_{\rm{log}}=\mathit{\exp}\left(\frac{2}{\lambda }{\int}_0^{\infty}\frac{d\omega}{\omega }{\alpha}^2F\!\left(\omega \right)\ln \omega \right)\!.\end{equation*}

Fitting the data presented in Carbotte [104], and Nicol and Carbotte [109] we obtained the following empirical linear relationship:

(5) \begin{equation*} \frac{2{\Delta}_0}{k_B{T}_c}=9.5\left(\frac{T_c}{\omega_{\rm{log}}}\right)+3.4. \end{equation*}

Substituting *T*_c_ = 300 K for a room temperature superconductor, ω_ln_ ≈ ω_opt_ ≈ 7*k*_B_*T*_c_, the estimated optimum frequency is 181 meV or 1137 cm^−1^. This value is not too far from the ‘best’ ω_opt_ of YH_6_ of 1200 cm^−1^.

**Figure 24.**
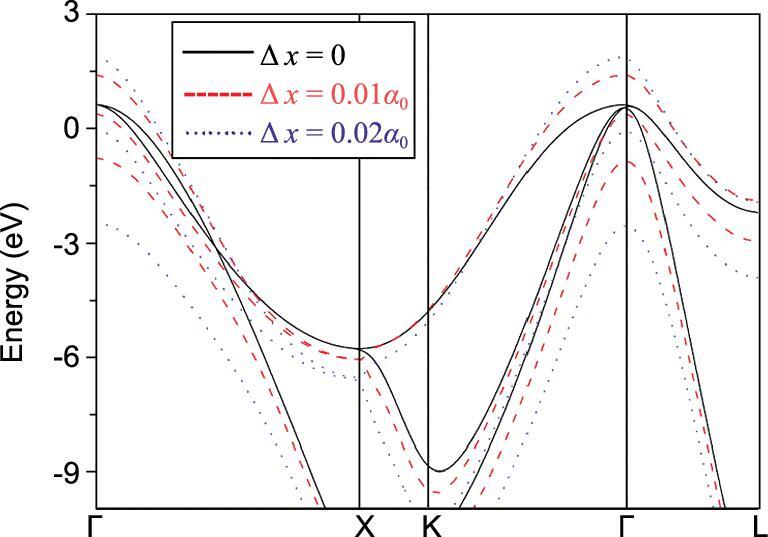
Change of the dispersion of the valence bands of B-doped diamond with respect to the amplitude of the displacement (Δ*x*) of the t_1u_ phonon mode (from Tse *et al.* [118]).

In a chemical description, large electron–phonon coupling corresponds to strong vibronic coupling, i.e. strong coupling between electronic and vibrational states [111–115] in molecules. It has been shown that vibronic coupling of molecules and solids shares many common features and, therefore, the consideration of this effect may help to design solids with strong electron–phonon coupling [115]. In molecular systems, the vibronic coupling is associated with the Jahn–Teller (JT) effect [116]: when the molecule is in a degenerate electronic state, it must distort to reduce the molecular symmetry and lower the total energy. For example, partial occupancy of a degenerate orbital will result in an orbitally degenerate electronic state, susceptible to Jahn–Teller (JT) distortion. Jahn–Teller distortion can be either static or dynamic in nature. A static distort results in a permanent change in the molecular symmetry. In a solid, the Jahn–Teller distortion is manifested as a structural phase transition associated with a lowering of the crystal space group symmetry. On the other hand, if the distortion is dynamic, temporal large amplitude normal mode vibrations lead to fluctuation of dispersion of the electron bands close to the Fermi level. The JT effect has been invoked to explain the superconductivity observed in boron (p-) doped diamond [117–119]. In diamond, at the zone centre the highest filled band in pure diamond is the majority C 2*p* triply degenerate t_1u_ crystal orbital [118]. In the rigid band model, the creation of ‘holes’ by replacing C with B (p doping) is equivalent to the removal of electrons from this band. As a result, spontaneous distortion must occur in order to alleviate the degeneracy. Figure 24 illustrates the electronic band structure of several small displacements of carbon atoms along the t_1u_ vibrational mode [119]. Even a small displacement of ∼0.03 Å (0.01*a*_0_, *a*_0_ is the equilibrium unit cell size) was found to effect large changes in the valence band dispersions close to the Fermi level, particularly at the zone centre (Γ) [119]. A similar explanation has been employed to rationalize the strong electron–phonon coupling and the very high *T*_c_ predicted for CaH_6_ [85]. Investigations into the interplay between the vibronic coupling and strong electron–phonon couplings in solids may provide further insight into this mechanism and contribute to developing strategies for synthesizing materials with even higher *T*_c_ [120].

A useful guide for the prediction of superconductivity is the theory of the coexistence of steep and flat bands in momentum space [121–126]. It is suggested that real space electron–hole pairing is intimately related to chemical bonding. A signature of strong interactions is the simultaneous presence of electron bands with vanishing Fermi velocities (flat bands) and high Fermi velocities (nearly free electron conduction band) near the Fermi level. The former can be considered as intermediate polaron–phonon coupling as in the high *T*_c_ cuprates [121,123]. The latter is the conventional weak coupling in the mean field theory of superconductivity. The two-band model [127,128] has been used successfully to explain the *T*_c_ in a variety of 2D layer compounds [126,128] including Ca [127], CaC_6_ [121] and MgB_2_ [127]. The coexistence of steep and flat bands has also been noted in the band structures of superconducting hydrides SnH_4_ [91], CaH_6_ [85] and H_3_S [129].

## THE ENERGY BARRIER

**Figure 25.**
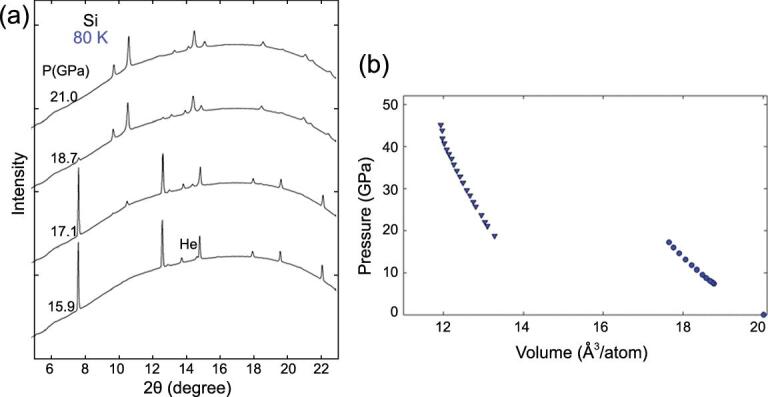
(a) Diffraction patterns of Si measured in a He pressure-transmitting medium at 80 K. (b) The abrupt volume collapse at 17 GPa (adapted from Tse *et al.* [50]).

In high pressure experiments, it is not uncommon that the thermodynamically stable crystal structure predicted by theory is not observed. In a structural transformation, several factors need to be considered such as the nature of the compression, deviatoric stress, surface energy of the sample, and the kinetics. The kinetic effect is governed by the energy barrier associated with the structural transformation. An example is the well-established transformation sequence in Si at low pressure. When compressed at room temperature, FCC Si (Si-I) transforms to metallic β-tin structure (Si-II) at 11 GPa, then to an orthorhombic structure (Si-XI) at 13 GPa, followed by a transition to a simple hexagonal phase (Si-V) at 16 GPa [130]. It is noteworthy that an earlier theoretical calculation [131] has shown that the transformation from Si-I and Si-II should not be direct as the total energy of Si-II is higher than that of Si-XI. It is even more surprising that when Si was compressed at low temperature (80 K) in a quasi-hydrostatic pressure-transmitting medium, a direct transformation from the Si-I to Si-V structure was observed at 17 GPa [50] (Fig. 25), bypassing the two thermodynamically stable intermediate Si-II and Si-XI [132] phases. The unexpected direct phase transition at low temperature is obviously kinetic in origin. Since the superconducting critical temperatures of the high pressure polymorphs of alkali and alkaline metals are very low, the unexpected finding in Si raises questions about the actual structure of the superconductivity phases and the low temperature structures of the complex phases discussed above.

**Figure 26.**
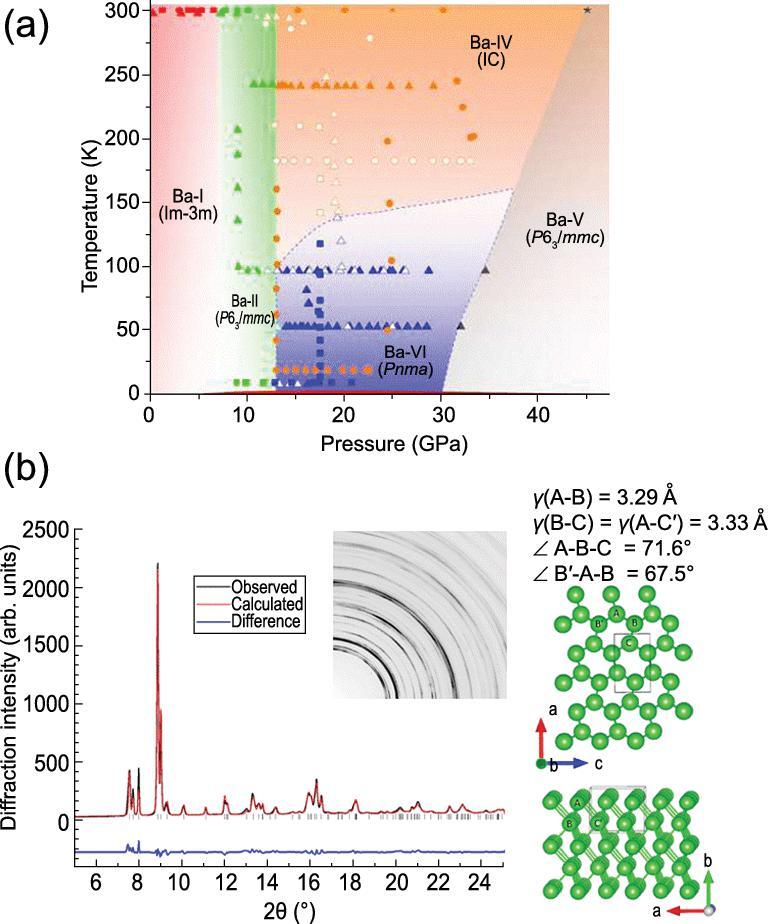
(a) The pressure–temperature phase diagram of Ba. (b) The low temperature orthorhombic structure of Ba (adapted from Desgreniers *et al.* [135]).

At ambient temperature [133], BCC Ba (Ba-I) transforms to a hexagonal close-packed (HCP) structure (HCP, Ba-II) at 6 GPa. Further compression to 12 GPa leads to the incommensurate (IC) self-hosting structure (Ba-IV) [132,133] similar to Rb-IV. Superconductivity with a *T*_c_ of ∼5 K was found above 14 GPa [134]. Is the superconductivity associated with the complex guest–host structure observed at room temperature? To investigate the temperature effect, the structural transformations of Ba were re-examined at low temperature and high pressure [135]. The results are summarized in the phase diagram shown in Fig. 26a. In one experiment, Ba was compressed at room temperature to 9 GPa. The known BCC → HCP transformation at 7 GPa was reproduced. The sample was then cooled to 9 K and compressed further. A structural phase transition was observed

at 14 GPa. This phase was not the complex IC phase. The new phase was stable up to 30 GPa where it transformed to Ba-V with the HCP structure. When heated at 18 GPa, the new phase transformed into IC Ba-IV at 130 K. The transformation was not reversible by cooling. In another *P*–*T* path, Ba-I was compressed at room temperature directly into Ba-IV. Cooling the sample at 20 and 30 GPa did not recover the new phase. It is obvious the new phase was meta-stable as its occurrence was dependent on the *P*–*T* path. This new phase (Ba-VI) was found to be orthorhombic and the structure closely resembled that of the precursor HCP Ba-II (Fig. 26b). The transformation from Ba-II to the IC Ba-IV involved substantial atomic displacement and atomic arrangement. Additional energy was required to overcome the energy barrier. At low temperature, the thermal energy was not sufficient to overcome the barrier and the Ba atoms adopted the lowest energy path and transformed to the distorted hexagonal structure. The new Ba-VI phase has been predicted to be a superconductor with a *T*_c_ of 3.6 K at 16.2 GPa [135]. A recent experiment confirmed the prediction by following the same low temperature *P*–*T* compression path. The sample was found to be a superconductor with a *T*_c_ of 3.5 K at 16.5 GPa [136], in remarkable agreement with the theory. In comparison, when Ba is compressed at room temperature and then cooled, it retains the IC Ba-IV structure, which is also found to be superconductive with a slightly lower *T*_c_ of 1.5 K at 15 GPa [134]. As alluded above, the lower *T*_c_ is likely associated with the fact that the transformation to the complex IC helps to open a gap in part of the Brillouin zone helping to lower the total energy of the system but not to the extent of becoming an insulator.

An unusual case of kinetic energy barrier is found in the recently reported superconductive dense H_2_S [9]. Apart from the perceived contamination from elemental S, attributed to decomposition, both the observed *T*_c_ and *in situ* diffraction pattern [107] are in good agreement with the predicted H_3_S [108]. It is now generally accepted that H_3_S is the superconducting phase. However, there are a few remaining problems. It is noted that superconductivity is observed only when H_2_S is compressed at low temperature along a particular *P*–*T* path [107]. When H_2_S was compressed following different thermodynamic paths, different X-ray diffraction patterns have been reported even at similar pressure–temperature conditions [137,138]. The diffraction pattern of H_3_S synthesized directly from a stoichiometric mixture of H_2_ and S also showed the two weak peaks [139] similar to the *in situ* diffraction pattern [107] that was assigned to S impurities. However, if H_2_S was decomposed to H_3_S and S, the intensity ratio of the two species was grossly inconsistent with the measured X-ray diffraction (XRD) pattern [129]. A plausible explanation is that the weak reflections in the XRD patterns may not have been S impurities. It is possible the superconducting phase may have been a metastable product.

**Figure 27.**
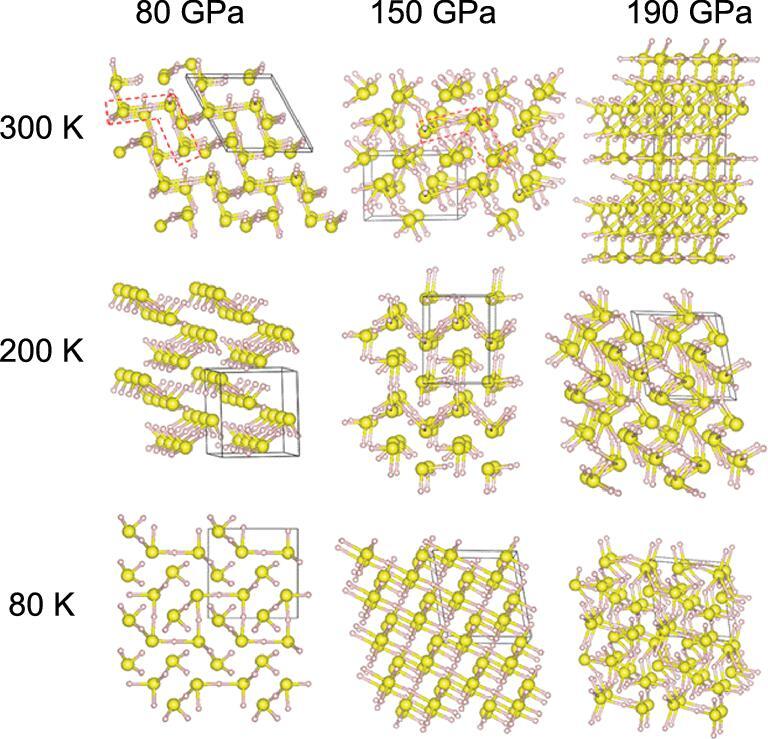
Crystal structures of H_2_S obtained from metadynamics simulations performed out at different pressures and temperatures, starting from the *Pmc*2_1_ structure. Large and small spheres represent S and H atoms, respectively. Unit cells are marked with black boxes (from Majumdar *et al.* [142]).

**Figure 28.**
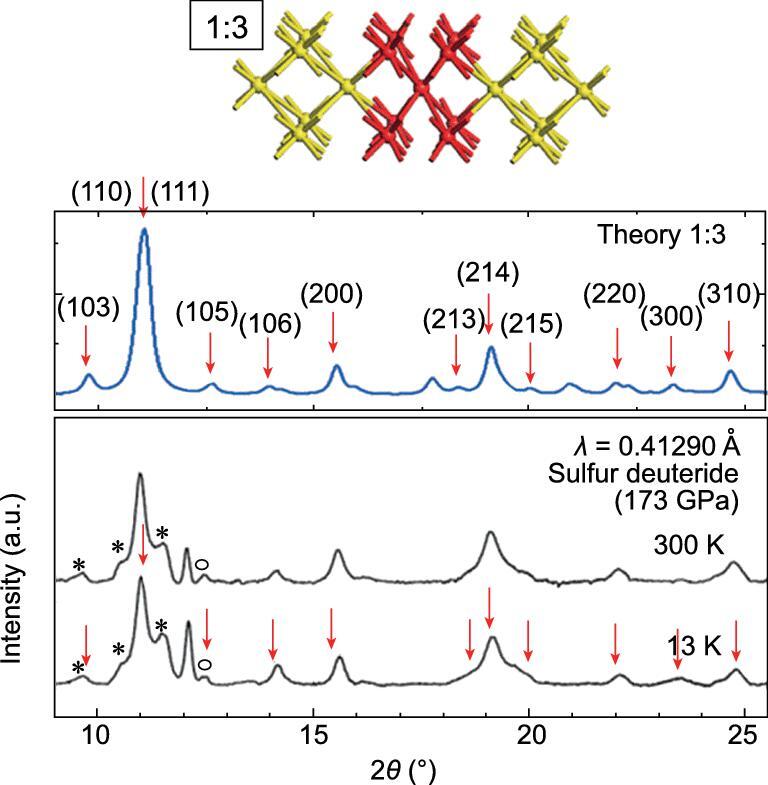
Comparison of the simulated XRD pattern of the 1:3 modulated structure with experimental diffraction patterns of high pressure H_2_S at 200 GPa (adapted from Majumdar *et al.* [141]).

To study the effects of temperature on the compression of H_2_S, metadynamics calculations [140] were performed [141,142]. In the experiment, H_2_S was initially compressed at 200 K then cooled and further compressed to 150 GPa. Superconductivity was observed upon warming. The calculations started with a structural model predicted to be stable at 80 GPa with the calculated diffraction pattern in good agreement with the experiment [143]. Simulations were performed at 80, 200 and 300 K. The metastable structures obtained at 80, 150 and 190 GPa are shown in Fig. 27. There are distinct differences in the structural motifs at the three different temperatures. At 200 GPa and 300 K, the H_2_S decomposed and a 3D framework was built solely of connected S atoms suggesting the onset of phase segregation. At 80 K and 80 GPa, the metadynamics simulation showed H_2_S self-ionized into (SH)^−^(H_3_S)^+^ with the SH forming zigzag chains and the S atoms situated at distorted BCC lattice sites, incidentally, similar to the S atom positions in the predicted H_3_S structure [108]. However, this structure has a *Pc* space group. Constant volume–constant temperature (*NVT*) and constant pressure–constant temperature (*NPT*) molecular dynamics calculations were performed on a supercell model constructed with S placed at the idealized BCC sites and the H atom positions taken from the *Pc* structure. In the *NVT* simulation, the S atoms vibrated about the BCC sites but the H atoms underwent rapid diffusions and exchanged between the SH^−^ and H_3_S^+^ moieties. Variable cell *NPT* simulation revealed a deformation of the BCC lattice [141]. Remarkably, a portion of the supercell was tetragonally distorted while the rest remained cubic. The equilibrium structure was a commensurate modulation of tetragonal and cubic regions alternated in a 1:3 ratio (Fig. 28). Incidentally, the calculated XRD pattern of the 1:3 modulated structure is in good accord with the experiment (Fig. 28). In particular, the two weak Bragg peaks previously assigned to S impurities were reproduced at the correct diffraction angles and intensities. At present, it is not feasible to compute the electron–phonon coupling of a fluxional and modulated system from First Principles electronic structure calculations. An order of magnitude estimate of the *T*_c_ of the modulated structure can be made from the Debye temperature (Θ*_D_*) derived from the theoretical vibrational density of states and using the McMillan equation [92]. Assuming λ between 1.0 and 3.0, which is within the range calculated for H_3_S, the *T*_c_ is estimated to be 107–221 K. These values are comparable to the experimental values [9]. Additional calculations starting from the low pressure crystalline phase show that the transformation to the high pressure-modulated structure is the result of a phonon instability near 150 GPa [142]. Furthermore, the calculated *T*_c_ of the low pressure structure is in good agreement with the experimental ‘low pressure phase’. Metadynamic calculations established a viable and consistent link on the evolution of the low pressure crystalline molecular phase to the high pressure structures, and explain the emergence and progression of superconductivity [142]. There is no need to invoke *ad hoc* assumptions of exotic intermediate ‘Magnéli’ phases [144] in which it is necessary that the S atoms be removed from the sample continually in order to transform eventually to the H_3_S phase. Consideration of a finite temperature effect by conventional molecular dynamics and metadynamics calculations clearly shows the structures obtained are dependent on the temperature and different from those predicted from total energy structural predictions (performed at 0 K). I recognize that the mechanical work can compensate for the strength of the S–H bond of 1.6 eV at 2.0 eV. However, one should also consider the energy needed to overcome the activation barrier in H–S bond breaking and the substantial rearrangement of the atoms in the new structure. Since the compression was performed at low temperature and the purported H_3_S structure does not possess any S–S bonds, additional energy is required to rearrange the atoms. This process is likely to be impeded by kinetics.

## OUTLOOK

In recent years, there has been a growing interest within the chemistry community to investigate the structures of high pressure solids and use of high pressure for chemical synthesis [5,145]. Development of chemical principles to understand the structure and chemistry at high pressure is beneficial to facilitate the research. On the other hand, high pressure chemistry is important for understanding the structure, properties and dynamics of the Earth’s minerals under high pressure and high temperature conditions. Recent theoretical studies have shown that the chemical principles under ambient conditions may not be directly transferable to extreme conditions. For example, calculations [146] have found that dissolved CO_2_ in water mostly exists as solvated CO_3_^2−^ and HCO_3_^−^ at 11 GPa and 1000 K. Moreover, ion pairing between Na^+^ and HCO_3_^−^ and CO_3_^2−^ ions is strongly dependent on the *P–T* condition. This effect may have a significant influence on the mechanism for transportation of carbon/carbonate species to the Earth’s mantle. A recent study has also shown that water can react with silica under very mild temperature and pressure conditions [147]. The water formed and trapped in the cavities created by decomposed SiO_2_ is highly acidic with a very high concentration of H_3_O^+^_._ Therefore, the aqueous chemistry is expected to be very different from that under normal conditions. It has also been found that the often-used structural rules for topological ordering in oxide solids and glasses based on the oxygen volume fraction [148] are no longer applicable under high pressure [149]. The continuous change in the electronic structure of the oxygen atoms under pressure affects the physical size of the atom and the assumption of a constant ratio of the ionic radii based on information at low pressure is not valid.

The orbital concept introduced here is very powerful. For example, contrary to second-row elements, the σ-bonded first row elements and their compounds are quite resilient to pressure. The reason is there are no low-lying orbitals available for these atoms to hybridize to change the nature of the chemical bond and the local geometry. For example, water can be compressed to 100 GPa without changing the tetrahedral oxygen environment even though several structural transitions have occurred. At high pressure, the local structures of boron in the oxide glass are very robust. The B–O coordination number (CN) changed initially from 3 to 4 under moderate pressure due to the change from *sp*^2^ to *sp*^3^ but no further change was observed up to megabar pressure [150]. Extension and improvement of the chemistry and bonding principles along this line of thought to elucidate novel phenomena observed at high pressure will be invaluable to the research.
